# A randomised controlled trial of an exercise intervention promoting activity, independence and stability in older adults with mild cognitive impairment and early dementia (PrAISED) - A Protocol

**DOI:** 10.1186/s13063-019-3871-9

**Published:** 2019-12-30

**Authors:** Rupinder K. Bajwa, Sarah E. Goldberg, Veronika Van der Wardt, Clare Burgon, Claudio Di Lorito, Maureen Godfrey, Marianne Dunlop, Pip Logan, Tahir Masud, John Gladman, Helen Smith, Vicky Hood-Moore, Vicky Booth, Roshan Das Nair, Kristian Pollock, Kavita Vedhara, Rhiannon Tudor Edwards, Carys Jones, Zoe Hoare, Andrew Brand, Rowan H. Harwood

**Affiliations:** 1 Divison of Rehabilitation, Ageing and Wellbeing, University of Nottingham, Nottingham, NG7 2UH UK; 2 School of Health Sciences, University of Nottingham, Nottingham, NG7 2UH UK; 3 Patient and Public Involvement Collaborator, Nottingham, UK; 4 Healthcare of Older People, Nottingham University Hospitals NHS Trust, Queen’s Medical Centre, Nottingham, NG7 2UH UK; 5 Mental Health Services for Older People, Nottinghamshire Healthcare NHS Foundation Trust, Highbury Hospital, Nottingham, NG6 9RD UK; 6 Institute of Mental Health, University of Nottingham, Nottingham, NG8 1BB UK; 7 Division of Primary Care, University of Nottingham, Nottingham, NG7 2RD UK; 8 Centre for Health Economics and Medicines Evaluation, Bangor University, Bangor, LL57 2PZ UK; 9 North Wales Organisation for Randomised Trials in Health Clinical Trials Unit (NWORTH CTU), Bangor University, Bangor, LL57 2PZ Wales UK

**Keywords:** Dementia, Cognitive impairment, Activities of daily living, Physiotherapy, Occupational therapy, Falls prevention, Tailoring, Strength training, Balance training, Dual-task training

## Abstract

**Background:**

People with dementia progressively lose cognitive and functional abilities. Interventions promoting exercise and activity may slow decline. We developed a novel intervention to promote activity and independence and prevent falls in people with mild cognitive impairment (MCI) or early dementia. We successfully undertook a feasibility randomised controlled trial (RCT) to refine the intervention and research delivery. We are now delivering a multi-centred RCT to evaluate its clinical and cost-effectiveness.

**Methods:**

We will recruit 368 people with MCI or early dementia (Montreal Cognitive Assessment score 13–25) and a family member or carer from memory assessment clinics, other community health or social care venues or an online register (the National Institute for Health Research Join Dementia Research). Participants will be randomised to an individually tailored activity and exercise programme delivered using motivational theory to promote adherence and continued engagement, with up to 50 supervised sessions over one year, or a brief falls prevention assessment (control). The intervention will be delivered in participants’ homes by trained physiotherapists, occupational therapists and therapy assistants. We will measure disabilities in activities of daily living, physical activity, balance, cognition, mood, quality of life, falls, carer strain and healthcare and social care use. We will use a mixed methods approach to conduct a process evaluation to assess staff training and delivery of the intervention, and to identify individual- and context-level mechanisms affecting intervention engagement and activity maintenance. We will undertake a health economic evaluation to determine if the intervention is cost-effective.

**Discussion:**

We describe the protocol for a multi-centre RCT that will evaluate the clinical and cost-effectiveness of a therapy programme designed to promote activity and independence amongst people living with dementia.

**Trial registration:**

ISRCTN, ISRCTN15320670. Registered on 4 September 2018.

## Background

People living with dementia experience progressive and irreversible loss of memory and other cognitive functions, which is severe enough to affect daily functioning [1–3]. Loss of ability occurs due to multiple factors, including cognitive impairment (memory loss, apraxia, executive dysfunction), dementia-related motor and balance impairment, physical co-morbidity, deconditioning and loss of opportunity [2–4]. Restricted opportunity is often due to concerns about safety, including risk of falling, and failure to adapt activities to residual or individual abilities [5, 6]. Mild cognitive impairment (MCI) comprises cognitive impairments that do not interfere with everyday activities, but which progress to dementia at the rate of about 10% per year [7, 8].

Moderate or vigorous intensity exercise, two to three times a week, improves strength, gait speed and performance in activities of daily living (ADLs) [9–13] and may slow cognitive decline [9, 12, 14–19]. Strength, balance and dual-task training can improve executive function, dual-task performance and gait parameters [20–25]. Functionally oriented therapy can improve ability in ADLs [15, 26–28]. Progressive strength and balance training in older people, with and without dementia, reduces risk of falling and reduces carer strain and it can also potentially benefit quality of life, mood and confidence [8–13, 29, 30].

FINALEX, a 12-month trial of an intensive, home-based, supervised exercise programme for people with dementia demonstrated a reduction in decline of ADLs and halved the rate of falling [31]. This study showed that, with the right support, people with dementia can achieve and sustain an intensity of exercise needed to gain health benefits. The challenge is how to achieve sufficient participation, adherence and persistence in routine health and social care services. Additionally it is necessary to establish how to translate individually tailored home-based programmes for people with dementia into sustainable programmes that are deliverable in the community.

A clinical academic team comprising academic researchers, occupational therapists, physiotherapists, rehabilitation support workers, health psychologists, nurses, geriatricians and Patient and Public Involvement (PPI) collaborators developed a complex intervention aimed at promoting activity and independence and reducing risk of falls for people living with MCI or mild dementia (the PrAISED intervention) [32]. The core principles of the intervention include 150 min of physical activity per week (including balance-challenging exercises), activities which are both active and functional, encouraging positive risk taking, facilitating community or environmental access and promoting independence [32]. The aim in intervening early is to enable people to ‘live well’ with dementia, prevent crises and establish positive health habits before the inevitable progression of dementia.

The feasibility and practicability of delivering and evaluating the intervention has been established in a prior study with 60 participants [33] (Goldberg et al., Promoting Activity, Independence and Stability in Early Dementia (PrAISED): a feasbility, multisite, randomised controlled trial, forthcoming). The feasibility randomised controlled trial (RCT) enabled refinement of mechanisms for tailoring the therapy programme to individuals’ needs and characteristics. Research procedures were updated for the present RCT to replace or omit measurement scales that did not perform satisfactorily, and to ensure that an informant was recruited for each participant. Additionally, we established that blinded ascertainment of outcome data was impossible, as inadvertent revelation of group allocation by participants occurred in more than 80% of cases, despite considerable efforts to maintain blinding [34].

Informed by results from the feasibility study, we now present a protocol for a definitive, multi-centred RCT (protocol version 2.3, 4th April 2019). The protocol has been extensively peer reviewed as part of the funding application process.

### Aim

The aim of the study is to determine the clinical and cost-effectiveness of a therapy programme to promote activity and independence, as well as stability, amongst people with early dementia and MCI.

## Objectives and methods

The study objectives are to:

- Determine whether the intervention reduces disability in ADLs; increases level of physical activity; improves balance, quality of life, mood and cognition; and decreases apathy, rate of falling, rate of hospital and care home admission, days spent in hospital and carer strain
- Determine if the intervention is cost-effective, within the trial period, over the anticipated remaining lifespan of participants, using a social return on investment model
- Evaluate the individual- and contextual-level mechanisms that contribute to study outcomes and evaluate the implementation of the therapy programme.

### Study design

**Fig. 1:**
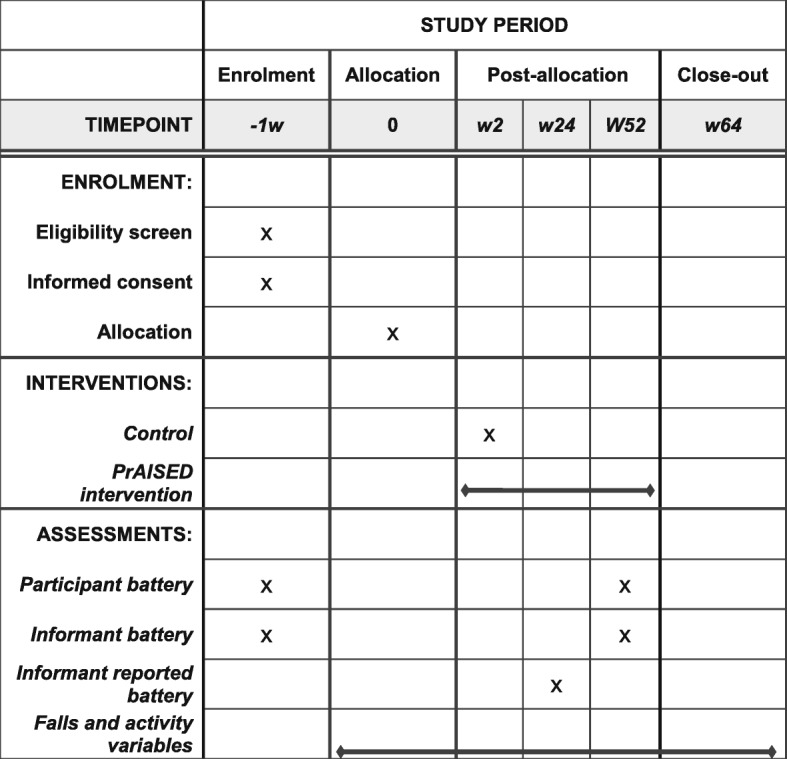
Schedule of enrolment, interventions and assessments

**Fig. 2:**
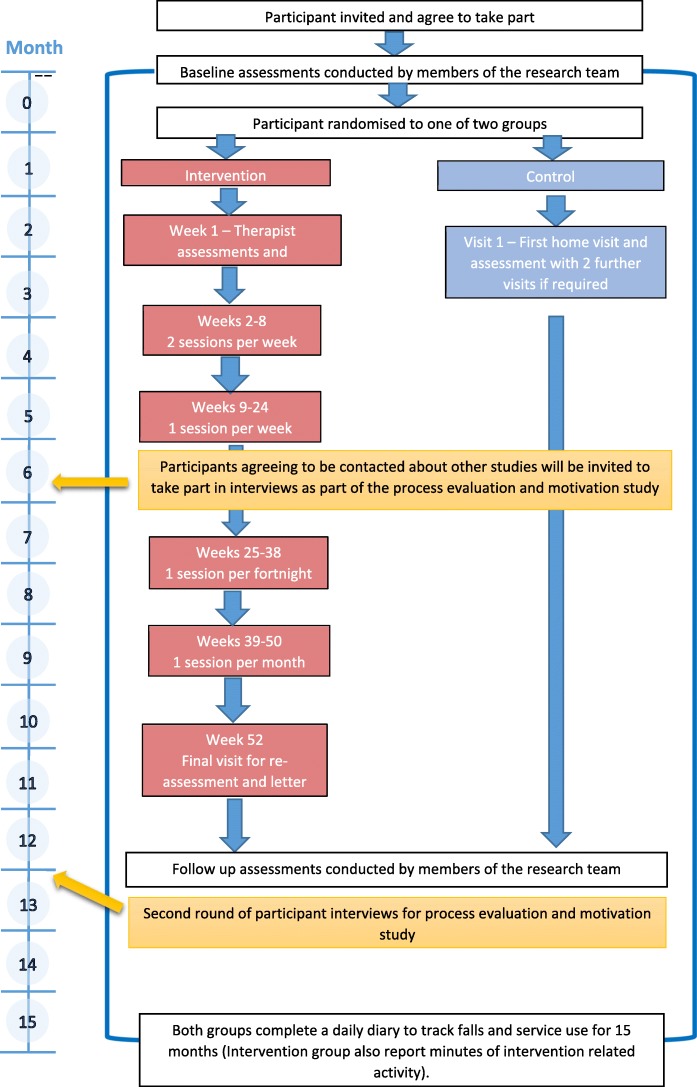
Overview of participant-related study processes (recruitment, assessment, intervention and interviews)

A multi-centre, individually randomised, pragmatic, parallel-group, controlled trial will be conducted (Fig. 1). Participants will give informed consent, and will be randomised to one of two arms: the intervention arm comprising supervised therapy, or the control arm, comprising falls risk assessment and advice (Fig. 2). Mixed methods will be used to undertake process evaluation [35] and economic analyses. The Standard Protocol Items: Recommendations for Interventional Trials (SPIRIT) checklist is provided as Additional file 1.

### Setting

Recruitment will be from locality-based, secondary-care memory clinics, general practice registers, dementia support groups and the National Institute for Health Research (NIHR) Join Dementia Research register. Intervention sessions will be one-on-one with the participant and therapist, delivered in the participant’s home or local community.

### Recruitment

The trial will recruit 368 participants and family or carer dyads from different sites in England. Recruitment will be carried out by a study researcher, who will assess mental capacity, take consent and collect baseline data from both members of the dyad.

### Participant inclusion/exclusion criteria

Participants will be eligible to participate if they meet the following criteria: are age 65 years or older; have a diagnosis of MCI or dementia (of any subtype except dementia with Lewy bodies); have a Montreal Cognitive Assessment (MoCA) score of 13–25 (out of 30); have a family member, informal carer or friend who knows the participant well (defined as having contact with them for at least one hour per week via Internet, telephone or in person) and who is willing and able to act as an informant and be a research participant. Participants with dementia/MCI must be able to walk without human help, communicate in English, be able to see and hear, have sufficient dexterity to perform neuropsychological tests, have the mental capacity to give consent to participate and consent to do so.

Participants will be ineligible to take part if they have a diagnosis of dementia with Lewy bodies, a co-morbidity preventing participation (e.g. severe breathlessness, pain, psychosis, Parkinson’s disease or other severe neurological disease) or they expect to be unavailable over the next year (e.g. due to plans to relocate or go on a long holiday, or a life expectancy of less than a year).

Participants will discontinue the intervention if they withdraw consent or if the therapist overseeing their care decides they are no longer able to take part (e.g. due to illness or injury, progression of their disease or inability to engage despite adjustment and tailoring of the programme). Participants who withdraw will be invited to remain in the trial for collection of outcome measures if willing. Withdrawn participants who have been randomised will not be replaced, as the sample size calculations make allowance for dropout. Abrupt termination of study treatment will not affect participant safety.

For the process evaluation [35], we aim to purposively select a subsample of five participants and their carers at each of the research sites. In addition, we will invite any participants who withdraw from the study to be interviewed, to explore their reasons for withdrawal.

### Randomisation

Participants will be individually randomised, stratified by site, the presence of a co-resident carer and history of previous falls, using a dynamic, adaptive allocation algorithm [36] accessed by a secure web portal to the system held at the North Wales Organisation for Randomised Trials in Health Clinical Trials Unit (NWORTH CTU), Bangor University. The randomisation system will be maintained by a statistician independent of the analysis and research teams. Access to the study website will be password protected. After randomisation and irrespective of intervention group allocation, a member of the local research team will inform the participant about the treatment and follow-up plan and inform the therapy provider so a visit can be arranged.

### Blinding

Blinding of intervention is impossible for participants and therapists administering the intervention. Blinding of researchers to allocation during outcome assessment proved impossible in practice during the feasibility study, as participants frequently inadvertently unblinded the researcher (Goldberg et al., Promoting Activity, Independence and Stability in Early Dementia (PrAISED): a feasbility, multisite, randomised controlled trial, forthcoming). This problem has also been reported in similar trials [34]. Researchers collecting baseline and outcome data will therefore not be blinded to allocation. To minimise ascertainment bias, we will use specific awareness training to avoid bias of researchers and collection of hospital and resource use data. We will use a ‘case manager’ system for recruitment and data collection (having a single person undertake all assessments and act as the main point of contact for a given participant), which proposes to improve support to participants, aiming to maximise retention in the study.

Statistical analysis will be blind to allocation.

### Baseline data collection

Researchers will visit participants and their informants in pairs, simultaneously interviewing the participants and informant dyads individually to complete assessments. Assessments will include (see Table 1):

1. Demographic and social variables
2. Medical and falls history, including previous fractures, hospitalisation and drugs taken
3. Frailty and falls (SHARE frailty assessment and falls diary) [50]
4. Blood pressure, sitting and standing (Omron M6 comfort (HEM-7321-E) automatic sphygmomanometer)
5. Cognition (MoCA, animal-naming verbal fluency task and Cambridge Neuropsychological Test Automated Battery) [48, 49]
6. Mood (Hospital Anxiety and Depression Scale and Apathy Evaluation Scale) [42, 43]
7. Activities of daily living (Nottingham Extended Activities of Daily Living questionnaire and Disability Assessment for Dementia [DAD]) [37, 38]
8. Physical activity (Longitudinal Study of Ageing Amsterdam physical activity questionnaire and Misfit Shine accelerometer) [44]
9. Quality of life (Dementia Quality of Life [DEMQOL], EuroQoL five-dimension three level [EQ-5D-3L] and EQ-5D-5L) [39–41]
10. Balance and mobility (Berg balance scale, Timed Up and Go, dual-task Timed Up and Go) [45–47]
11. Health and social care resource use (Client Service Receipt Inventory [CSRI]) [51]
12. Carer strain (Caregiver Strain Index), carer health-related quality of life (EQ 5D-5L), participant service use (CSRI) [51, 52]
13. Personality (Big Five Inventory-10) [53].

**Table 1:** Health status outcomes measured at baseline and after 12 months

Outcome	Measured by	Respondent
Activities of daily living	DAD [37]	Informant
	NEADL [38]	Patient
Quality of life	EQ-5D-3L self [39]	Patient
	EQ-5D-5L proxy [39]	Informant
	DemQOL [40, 41]	Patient
	DemQOL proxy [40, 41]	Informant
Depression, anxiety and apathy	HADS [42]	Patient
	AES [43]	Informant
Physical activity	LAPAQ [44]	Informant
	Pedometer/accelerometer	Patient
Balance and mobility	Berg balance scale [45, 46]	Patient
	TUG [47]	Patient
	Dual-task TUG [47]	Patient
Cognition	CANTAB [48]	Patient
	MoCA [49]	Patient
	Verbal fluency (animal-naming task)	Patient
Falls, fractures and frailty	Diary	Patient and informant
	SHARE frailty assessment [50]	Patient and informant
Admissions to and days spent in hospitals and care homes	Diary	Patient
	Hospital records	
NHS and social service costs	CSRI [51]	Informant
Carer strain	CSI [52]	Informant
Carer health-related quality of life	EQ-5D-5L self [39]	Informant

*DAD* Disability Assessment for Dementia, *NEADL* Nottingham Extended ADL scale, *EQ-5D-3L* EuroQoL five-dimension, three-level (self), *EQ-5D-5L* EuroQoL five-dimension, five-level (proxy), *DEMQOL* and *DEMQOL proxy* Dementia Quality of Life, *HADS* Hospital Anxiety and Depression Scale, *AES* Apathy Evaluation scale, *LAPAQ* Longitudinal Ageing Study Amsterdam Physical Activity Questionnaire, *Pedometer* Misfit Shine 2, *TUG* Timed Up and Go, *CANTAB* Cambridge Neuropsychological Test Automated Battery, *MoCA* Montreal Cognitive Assessment, *CSRI* Client Service Receipt Inventory, *CSI* Caregiver Strain Index, *NHS* National Health Service

### Intervention

The trial will compare the active and control interventions. The development and content of the programme has been published [32].

### Active intervention

The intervention programme will be tailored to individual abilities, co-morbidities, interests and goals. The intervention includes progressive strength and balance exercises; functional activities and risk analysis, training and advice; and environmental assessment and dual-task training, delivered using a motivational approach [32]. We will use a specially developed stratification tool to determine the frequency of intervention sessions for each participant to enable them to sustain the programme. Participants will receive between 9 and 50 visits from the trained study therapists and rehabilitation support workers over 52 weeks to directly supervise exercise, assess progress and provide progressively challenging activities and exercises. The programme is tapered to encourage habit formation and the continuation of self-directed exercise and activity between supervised sessions and after the programme has been completed. An option to pause or suspend the programme will be allowed to account for individual circumstances (such as holidays, illness or bereavement).

Participants will be encouraged to perform their intervention programme for at least 3 h per week. Previous guidance suggests at least 150 min of physical activity to promote healthy ageing [54] and at least 180 min of balance-challenging and progressive strengthening exercise per week to prevent falls [55]. A similar intensity has also been shown to improve neuropsychological function [25]. Spouses, partners, family members or carers are encouraged to support or participate as each individual circumstance allows. To encourage persistence after the intervention sessions and regular supervision ceases, participants will be introduced to local exercise, activity and community classes.

### Control

The control intervention has been modelled on usual care, consisting of a fall prevention assessment and follow-up. Control participants will receive an initial therapy visit and assessment within 2 weeks of the research assessment (baseline). This visit will be 90 min in duration. A further two visits are permitted (each of 90 min) by either the assessing therapist or rehabilitation support worker in order to review actions or expand on any advice provided following the falls assessment (for example, to seek a medicine review with the participant’s general practice physician). The advice and recommended actions are prompted by the falls assessment [56] and include actions for the participant (e.g. wearing more suitable footwear), the therapists (e.g. referral to local services) or both. Each visit received by the control participant will be 90 min in duration and will be completed within 3 months. The control participants are seen by the same group of therapists who administer the intervention.

### Concurrent treatment

All other interventions will be permitted, in both study arms, including cognitive stimulation therapy, use of acetylcholine inhibitor or memantine drugs or referral to mental health, medical, rehabilitation or falls prevention services. There are no arrangements for ancillary or post-trial care beyond routine clinical care. There are no special arrangements for compensation for non-negligent harm resulting from trial participation.

### Follow-up and outcomes

Each participant will take part for 15 months. After 6 months, informants will be asked to complete the EQ-5D-5L proxy [39], DEMQOL-U items from the DEMQOL measure [41] and the Short CSRI [51] about the participant, via post, with telephone calls to prompt or support as needed. The main follow-up is after 12 months (+/− 4 weeks), when two researchers will visit the dyads at home to collect outcome data by interviewing the participant and informant separately (Table 1). In the feasibility study, the follow-up rate at 12 months was 80% without differential loss between arms (Goldberg et al., Promoting Activity, Independence and Stability in Early Dementia (PrAISED): a feasbility, multisite, randomised controlled trial, forthcoming).

Participants will keep a monthly diary for falls and service use between months 0 and 15, with telephone calls to prompt or support as needed. Therapists delivering the intervention sessions will not be permitted to use or prompt the use of the monthly diary for either study arm.

For falls, the incidence rate ratio will be compared for the intervention and control groups. For all other outcome measures, the standardised effect size estimate (*d*) of the difference between the groups’ follow-up scores will be based on analysis of covariance (ANCOVA), which will take into account the baseline scores on the measures. Data will be aggregated using mean score unless descriptive statistics show the data to be substantially skewed or non-normal.

### Falls, programme adherence and service use

Adherence to the PrAISED programme, occurrence of falls and health and social care service use will be collected by monthly self-completed diaries. The nominated carer will be asked to prompt and oversee this task. Telephone calls will be made to prompt or support as needed.

### Process evaluation and motivation study

The process evaluation of the trial will follow Medical Research Council guidelines for the process evaluation of complex interventions [57]. It will be used to gather data around the implementation of the intervention, the mechanisms of impact and contextual factors and participants’ motivations [35].

The process evaluation will answer the question ’How does the intervention work?’ by identifying the mediating factors that contribute to study outcomes. It will use a mixed methods design and will comprise two studies. The implementation study will examine the training provided to therapists and how the intervention is delivered to participants, by looking at four domains:

- Fidelity (the consistency of training and delivery of the intervention)
- Adaptations (alterations made to training and delivery, to achieve better contextual fit)
- Dose (how much training and intervention is delivered)
- Reach (the extent to which therapists and participants come into contact with the intervention).

The study on the mechanisms of impact and context will focus on the individual- and context-level mechanisms that contribute to study outcomes. These will be investigated through semi-structured interviews with participants and carers from each site. To ensure that the full range of perspective is gathered, we will purposively recruit participants with dementia/MCI from four different groups:

- Low adherers to the PrAISED programme (i.e. participants who have undertaken less than 150 min of physical activity per week on average before the first set of interviews at month 6, as recorded on the calendar)
- High adherers to the PrAISED programme (i.e. participants who have undertaken more than 150 min of physical activity per week on average before the first set of interviews at month 6, as recorded on the calendar)
- Those who self-withdraw
- Participants from the control group.

Clinicians will also be interviewed about their experiences of receiving training and delivering the intervention. Details of the process evaluation can be found in the published protocol [35].

Data from the qualitative interviews of the process evaluation will be used to investigate motivational factors linked to adherence.

### Economic evaluation

We will determine if the intervention is cost-effective, by comparing utilities derived from EQ-5D and DEMQOL-U, with costs. Costs will be separately determined from health service, public-sector multi-agency and private or informal care perspectives. We will consider time frames of the trial period, and the anticipated remaining lifespan of participants, using Markov modelling. We will also use a social return on investment model [58]. These analyses will be subject to a separate protocol.

### Adverse events

Adverse event (AE) monitoring will begin when a participant has been randomised and will continue for 15 months. We will record AEs which are defined as serious (involving death, life-threatening events, hospitalisation, significant disability or incapacity) or which are potentially related to the intervention or to exercise undertaken independently. Inter-current illness, progression of dementia, loss of function, hospitalisation and death are likely to be common in this population given their age and frailty. We will assess all reported AEs for severity and relatedness to the intervention.

The intervention group will have more contact with professional staff than the control group, and therefore greater opportunity to report AEs, presenting a potential bias. We will screen falls diaries and medical records to ascertain core AEs across both arms. For purposes of comparing the safety of the intervention, we will consider ’deaths’, ’hospital admissions’ and ’falls’ to be core AEs. These will be ascertained and analysed as part of the core research dataset, independently of incidental reporting, and should therefore be more robust to information bias.

### Data management

A data management plan will be drawn up by NWORTH CTU as part of the trial setup. Data will be entered locally using the MACRO data entry system. For each time point and for each site, 5% of the cases entered into MACRO will be checked against the data recorded on the paper case report forms. All study co-applicants and researchers will have access to the final trial dataset. There are no contractual agreements limiting access for investigators.

### Statistics and data analysis

A Statistical Analysis Plan will be agreed between a CTU trial statistician, a second statistical advisor (based at the CTU) and the Chief Investigator, before completion of data collection. The Data Monitoring and Ethics Committee (DMEC) and the Programme Steering Committee (PSC) will approve the Statistical Analysis Plan.

Analyses will be undertaken by the CTU and will be performed blind to group allocation.

The first 50 randomised participants will constitute the sample for an internal pilot, to check on recruitment and data completion. There will be no other interim analyses.

### Sample size and justification

The primary outcome is the Disability Assessment for Dementia (DAD) at 12 months [37]. A sample size of 184 participants per group, including 23% attrition (based on previous studies [10, 31]), has 80% power to detect changes in the disability outcome, DAD, with effect size 0.5 (11 points on a baseline of 70, standard deviation 22, data from [10, 13]). This sample size has been rechecked using data from the feasibility study and confirmed to be a conservative estimate.

### Baseline comparison

Baseline variables will be compared across groups using means, medians or proportions as appropriate.

### Assessment of efficacy

Analyses will be conducted on an intention-to-treat basis. Missing data for outcome measures will be imputed, using multiple imputation, if more than 5% of the data are missing and data are shown to be missing at random.

We will compare the DAD scores between the intervention and the control groups, adjusting for stratifying variables and any baseline imbalances in an ANCOVA model. Adjusted mean differences between groups and standardised effect size estimates (equivalent to Cohen’s *d* [59]) will be calculated.

Rate of falls will be compared between the intervention and the control groups using the incidence rate ratio, from a negative binomial regression model, adjusted for baseline imbalance or variables with prognostic importance [60, 61].

Other secondary outcomes will be compared between the intervention and the control groups using ANCOVA to adjust for baseline imbalance or variables with prognostic importance.

### Dissemination

Results will be disseminated via a peer-reviewed report to the funder, which will be freely available, and through open access journal articles and conference presentations. We will inform participants of the results through a newsletter. Standard journal authorship criteria will apply; there will be no use of professional writers. Public or professional access to the full protocol, participant-level data and statistical code, for bona fide academic purposes, will be by request to the Chief Investigator.

### Patient and Public Involvement (PPI)

The study team includes two patient and public collaborators who have been involved in the development of the intervention, trial and additional evaluations. The PPI contributors worked with the study team to design participant facing materials (see Additional files 2, 3 and 4 for study consent form and participant information sheets) and contributed to the study design and management. The PPI contributors are co-researchers for the process evaluation, motivation and adherence study and will work with the research team to develop the interview topic guides, interview participants, analyse data and disseminate findings (e.g. attending conferences, delivering lectures and seminars and co-authoring papers for publication). Two external PPI representatives sit on the PSC.

### Governance

An independent PSC and DMEC will meet six-monthly in accordance with NIHR procedures. The trial sponsor is Research and Innovation, Nottingham University Hospitals National Health Service (NHS) Trust, NG7 2UH. Any decision to terminate the trial will be taken on the advice of the PSC, in conjunction with the sponsor and funder. Site visits will be undertaken every 6 months by co-ordinating centre researchers to audit compliance with trial procedures. Protocol changes will be communicated to sites within 35 calendar days in accordance with NIHR Clinical Research Network procedures.

## Discussion

We developed a complex intervention, and successfully conducted a feasibility RCT to demonstrate the practicability of its delivery, and of undertaking research procedures in this population and setting [32, 33]. We now present the protocol for a multi-centre RCT to determine its clinical and cost-effectiveness.

Dementia is progressive, affecting memory, other cognitive abilities and motor performance [4–6, 62]. These impairments impact an individual’s abilities to perform day-to-day activities and are linked to an increased risk of falls [4, 6]. Falls and related injuries lead to immobility, inactivity, loss of independence and increased dependence [62]. Early interventions aimed at maintaining activity and independence, which are specifically designed for individuals with dementia, may enable people to live well, prevent crises and reduce dependency. Exercise, strength, balance and dual-task training may have positive benefits, including improvements in gait speed and performance of activities of daily living, slowed cognitive decline and improved mood and confidence, whilst reducing the risk of falls [9–14, 29, 30].

People with MCI and mild dementia retain functional ability and independence, but are at high risk of progression and deteriorating health status. The range of neuropsychological deficits in MCI is similar to that in dementia [4, 63], and the distinction between the two is arbitrary (depending on the difficulty of daily activities considered). This provides the justification for considering the two conditions together. Pharmacological interventions are of limited efficacy in improving outcomes or slowing progression in these conditions [64]. New learning and adaptation are possible, but many interventions in dementia have proven ineffective when subject to rigorous evaluation. However, there are precedents for demonstrating effectiveness [24, 25, 27, 65]. The intervention that we describe is complex, reflecting the need to address multi-faceted problems, individual variation and real-world clinical practice. Our evaluation uses mixed research methods, to demonstrate clinical and cost-effectiveness, but also to understand how the intervention operates, what influences motivation and engagement and the necessary conditions for success and for potential future implementation.

Research involving people with cognitive impairment can be difficult, requiring adaptation and attention to their particular needs. Participants are potentially vulnerable, and thus careful, ongoing attention to the assessment of mental capacity and consent to participate is required. Progressive memory impairment challenges assessment of aspects of health status and ascertainment of healthcare and social care resource use, requiring the involvement of an informant. Experience of both ill health and healthcare includes multiple stakeholders, especially family carers, whose perspective must be included, requiring them to be included as research participants.

Recording of activity and falls through self-completion of diaries can be challenging, but we demonstrated in the feasibility trial that involving a family member and providing regular postal reminders can be effective mechanisms to aid participants to record such information (Goldberg SE, et al., ‘Promoting Activity, Independence and Stability in Early Dementia (PrAISED): a feasibility, multisite randomised controlled trial’; in preparation). High-level outcomes, such as quality of life, have multiple influences, including co-morbidities and socio-economic factors, which may not be amenable to therapeutic intervention. Complex interventions require measurement of a battery of outcomes, both to describe the impact of an intervention and to understand its mechanism, at the risk of type I statistical error through multiple comparisons. We paid particular attention to measures to achieve engagement and sustained adherence to the intervention over a long period of time, which appeared to be successful in the feasibility study.

In the feasibility RCT we found that outcome assessor blinding was not possible (Goldberg SE, et al.; in preparation). Previous studies have reported similar challenges to outcome assessor blinding in trials of home-based exercise interventions for older people [34]. We decided that outcome assessors will not be blinded to intervention in this trial, but to mitigate the potential bias this may introduce, all researchers conducting outcome assessments will complete specific anti-bias training.

This RCT is part of a research programme aimed at understanding activity limitation amongst people with dementia, how to intervene to promote activity and prolong independence and also how to get such a therapy programme to work in practice. The RCT opened to recruitment in October 2018 and will close recruitment in March 2020.

### Trial status

The trial opened to recruitment in October 2018 and will close to recruitment in March 2020 (protocol version 2.3 4th April 2019).

## Supplementary information

**Additional file 1.** SPIRIT 2013 checklist: recommended items to address in a clinical trial protocol and related documents.

**Additional file 2.** Participant consent form.

**Additional file 3.** Participant information sheet.

**Additional file 4.** Summary participant information sheet.

## Data Availability

Access to the full protocol, trial dataset and statistical code will be made available upon request to the Chief Investigator.

## Abbreviations

ADL: Activity of daily living
AES: Apathy Evaluation Scale
CANTAB: Cambridge Neuropsychological Test Automated Battery
CSI: Caregiver Strain Index
CSRI: Client Service Receipt Inventory
DAD: Disability Assessment for Dementia
DEMQOL: Dementia Quality of Life
EQ-5D-3L: EuroQoL five-dimension three level (self)
EQ-5D-5L: EuroQoL five-dimension five-level (proxy)
HADS: Hospital Anxiety and Depression Scale
LAPAQ: Longitudinal Ageing Study Amsterdam Physical Activity Questionnaire
MCI: Mild cognitive impairment
MoCA: Montreal Cognitive Assessment
NEADL: Nottingham Extended ADL scale
NHS: National Health Service
NIHR: National Institute for Health Research
PPI: Patient and Public Involvement
PrAISED: Promoting Activity, Independence and Stability in Early Dementia
RCT: Randomised controlled trial
SHARE: Survey of Health, Ageing and Retirement in Europe
TUG: Timed Up and Go
