# Supplementary material for: A randomised controlled trial of an exercise intervention promoting activity, independence and stability in older adults with mild cognitive impairment and early dementia (PrAISED) - A Protocol
Source: Trials. 2019 Dec 30;20:815. doi: 10.1186/s13063-019-3871-9 (PMC6937783; doi:10.1186/s13063-019-3871-9)
Supplement: Supplementary file 2 — Additional file 2. Participant consent form. [file 13063_2019_3871_MOESM2_ESM.doc]

**Use local headed paper**


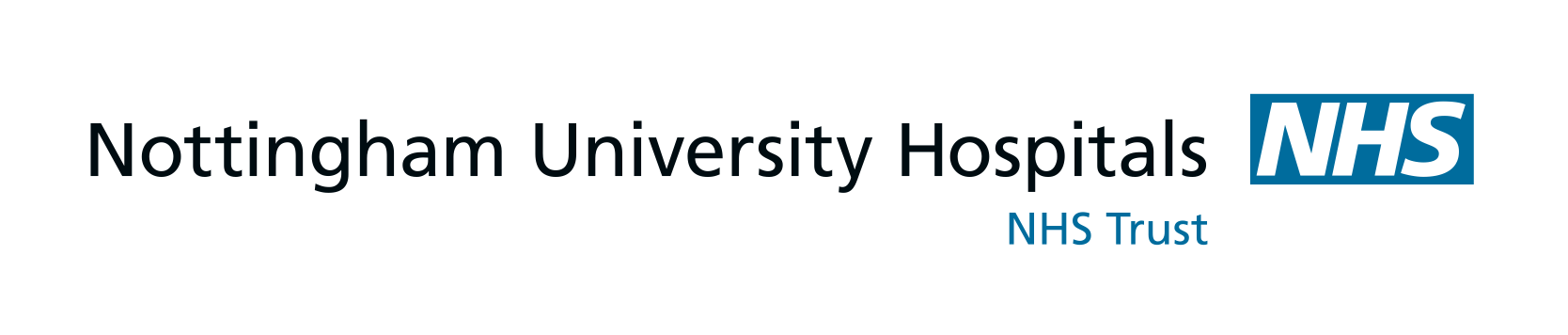

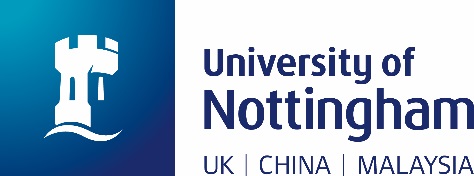


**PrAISED 2 Consent Form Patient**

Version: 2.1 13-11-18

**Promoting Activity, Independence and Stability in Early Dementia and Mild Cognitive Impairment (PrAISED 2)**

**Principal Investigator:** [SITE PI NAME HERE]

**IRAS Project Identification Number:** 236099

Patient Study ID: _ _ _ _ Patient initials:…………

The participant should initial each box if in agreement.

Initial here:

| 1. I confirm that I have read and understand the information sheet dated 13^th^ November 2018 (version 2.1) for the above study and have had the opportunity to ask questions. |  |
| --- | --- |
| 1. I understand that my participation is voluntary and that I am free to withdraw at any time without my medical care or legal rights being affected. |  |
| 1. I understand that my medical and research records may be looked at by authorised individuals from the Sponsor for the study and the UK Regulatory Authority in order to check that the study is being carried out correctly. |  |
| 1. I understand that even if I withdraw from the above study, the data collected from me will be used in analysing the results of the study, unless I specifically withdraw consent for this. |  |
| 1. I agree that my GP, or any other doctor treating me, will be notified of my participation in this study. |  |

| 1. I agree that the research team can access my health and care records to collect information for the research.   Initial here: |  |
| --- | --- |
| 1. *(Optional) I give permission for the research team to contact me to discuss other related studies.* |  |
| 1. *(Optional) I consent to sessions between myself and the therapist (for example, a physiotherapist) being video recorded.* |  |
| 1. I consent to the storage including electronic, of personal information for the purposes of this study. I understand that any information that could identify me will be kept confidential and that no personal information will be included in the study report or other publication. |  |
| 1. I agree to take part in the study |  |

Name of patient: Date: Patient’s signature:

Name of researcher: Date: Researcher’s signature:

**When completed:** 1 to be kept in care record, 1 copy for patient and 1 copy for researcher site file.
