# Supplementary material for: A randomised controlled trial of an exercise intervention promoting activity, independence and stability in older adults with mild cognitive impairment and early dementia (PrAISED) - A Protocol
Source: Trials. 2019 Dec 30;20:815. doi: 10.1186/s13063-019-3871-9 (PMC6937783; doi:10.1186/s13063-019-3871-9)
Supplement: Supplementary file 3 — Additional file 3. Participant information sheet. [file 13063_2019_3871_MOESM3_ESM.doc]

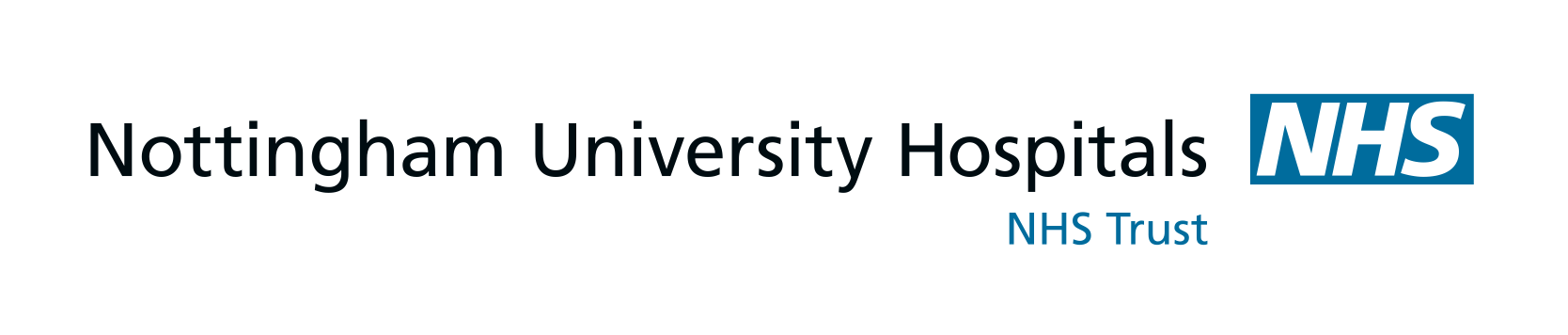

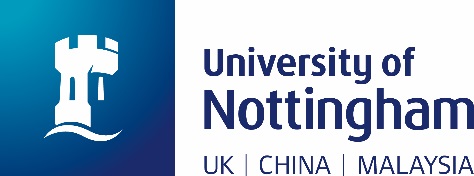


**Participant Information Sheet – Patient.**

**Title of Study:** Promoting Activity, Independence and Stability in Early Dementia and Mild Cognitive Impairment (PrAISED 2)

**Principal Investigator:** [Site PI name here]

**IRAS Project Identification Number:** 236099


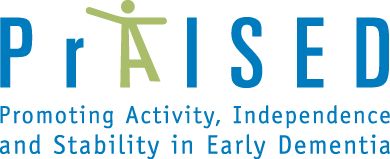

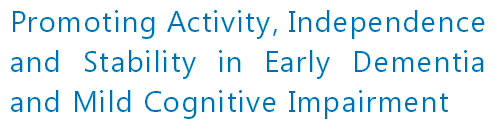


We would like to invite you to take part in a research study. Before you decide, you need to know why we are doing the research, and what it will involve. Please take time to decide if you want to take part or not. Taking part may involve a lot of time, effort and commitment, for at least a year. Please only volunteer to take part if you think you can finish the programme once you start. We will try to make your involvement worthwhile and interesting.

One of our team can go through the information sheet with you and will answer any questions you may have. You can also talk to others about the study if you wish.

**Why are we doing the study?**

People with memory problems can struggle with everyday activities. They may stop doing things they want to do. They are more prone to accidents and have a higher risk of falling. Occupational therapists can advise on how to do daily activities set at the right level, and safely. Physiotherapists can teach exercises, which improve balance, and increase activity, confidence and energy, and may also help maintain memory. The best results come from doing exercises several times a week for at least 6 months.

There is little research on how to make these therapies work for people with memory problems. In this study, we want to compare two different approaches.

**Why have I been invited to take part?**

You have been invited because you attended the Memory Assessment Service (Memory Clinic), because you registered with ‘Join Dementia Research’, or because you have been told you have dementia or ‘mild cognitive impairment’ by a medical professional.

**Do I have to take part?**

No. It is up to you whether you take part or not. If you decide to take part we will ask you and a family member or carer to sign a consent form to say that you agree, and understand what is involved. You are free to leave the study at any time without giving a reason. If you decide not to take part, or if you withdraw, this will not affect any other care you receive.

**What will happen to me if I take part?**

At the start, we will visit you and a family member or carer at home. We will ask questions about your daily life, health and wellbeing. There will be some physical assessments and tests of memory and thinking. If you agree to join, a computer will allocate you at random to one of two therapy possibilities; you do not get to choose.

A physiotherapist and occupational therapist will visit to make an initial assessment, give advice, and make a treatment plan.

You will be asked to complete a daily calendar to record any falls and activities for 15 months. We will give you a pedometer (a device which measures how much you walk) to wear for a week at the start and end of the study. We may telephone you to remind you to return your calendar each month. After six months we send a questionnaire by post. After a year, we will visit you again to ask questions about your health, abilities and wellbeing.

We have provided a diagram at the end of this information sheet for a summary of what is involved.

You may be invited to take part in other aspects of the research study which are optional. This may include an interview to see what you think of the therapy, or an MRI scan. We may want to video record you taking part in some of the therapy sessions. These videos would be used for training and research purposes.

**What are the therapy packages?**

Both packages include an assessment by qualified therapists (physiotherapists and occupational therapists). They will assess how well you can perform daily activities, your strength and balance, your home environment, and if there are things you want to do that you can’t. We will encourage you to try to do some exercises three times a week.

Therapy Package 1

If you are allocated therapy package 1, you will be given the standard NHS advice for anyone who is at risk of falling. An occupational therapist and/or physiotherapist will visit you to do an assessment, and give you falls prevention and exercise advice. You will be encouraged to follow this for as long as you are able. They will do up to two follow-up visits if they feel it necessary.

Therapy Package 2

If you are allocated therapy package 2, the therapists will develop a programme of activities and exercises with you and your family or carer, matched to your abilities, interests and goals. The details about your therapy programme will be written down and left with you in a home file for you to keep. You will have regular visits from an occupational therapist, physiotherapist and a trained rehabilitation support worker, who will come to your home, to help you do the exercises and adjust the programme. The therapists will always agree the time and date of visits with you. Visits will be frequent at the beginning of the programme, and reduce as the year goes on. The number of visits will depend on your needs and preferences, but will range from 9 to 50 visits over one year.

**Are there any possible side effects of the therapy?**

Activities and exercises are a standard part of routine occupational therapy and physiotherapy, and should be low risk. If you are not used to doing exercise, some muscle stiffness and fatigue is possible, especially in the early stages. If you are doing new activities or become more active, there is an increased risk of accidents such as falls, which could result in injury. Possible consequences could be bruises, sprains or fractures. It is unlikely that this will happen just due to the therapy package alone. Some conditions such as arthritis or angina may be made worse by exercise. The therapists will take account of this, and adapt what they ask you to do, so it is achievable.

Please report any problems you have to one of our researchers or therapists. We will ask you to record problems on a calendar. There is also a contact number given at the end of this information sheet for you to phone if you become worried at any time. In the unlikely event of a medical emergency occurring during therapy or research visit, we may contact a family member or care.

**Are there any other risks or disadvantages of taking part?**

Depending on which therapy package you receive, there may be a number of therapists visiting you. Participation in the therapy and research will take effort, commitment and time, but we will always visit at times to suit you. If we think that an activity you usually do is putting you at great risk, we may recommend you stop it. You and your family must decide if you want to follow any advice given, but you may find this guidance unwelcome.

**What are the possible benefits of taking part?**

Exercise is generally known to be beneficial to health and well-being, including benefits to heart, blood pressure, diabetes, joints, mood and daily life. You may find that you are better able to do your daily activities. You may enjoy having the researchers and therapists coming to visit you. Some people appreciate having the opportunity to contribute to the well-being of others through research. This activity and exercise package is currently being researched, and at this stage, we do not know if it will benefit you personally.

**What happens when the research study stops?**

Once the research stops, we will not be providing any more therapy visits or advice. However, before the research ends, we will work with you to find ways that you can continue with your exercises and activities on your own, or using community facilities such as leisure centres or classes.

**What if there is a problem?**

If you have a concern about any aspect of this study, please speak with the researchers or therapists visiting you, who will do their best to answer your questions. Alternatively, you can contact the chief investigator. Their contact details can be found at the end of this information sheet.

If you remain unhappy and wish to complain formally, you can do this through the NHS Complaints Procedure. Please contact the [Name of Local NHS Trust] Patient Advice and Liaison Service (PALS), on [Local PALS telephone number], or email [Local PALS email address]. You can also write to: [PALS postal address].

In the event that something does go wrong and you are harmed during the research there are no special compensation arrangements. If you are harmed due to someone’s negligence, you may have grounds for legal action for compensation, but you may have to pay your legal costs. The normal NHS complaints mechanisms will still be available to you.

**Will my information be kept confidential?**

Yes. All the information about you and your participation in this study will be kept confidential. You will be given a study number, which will be used as a code to identify you on all study forms. The information will be held securely on paper and on computers at the Universities of Nottingham, Bangor and [ENTER SITE HERE], under the provisions of the General Data Protection Regulation 2018. For more information on how your data will be processed please refer to the Privacy statement at the end of this sheet.

Professional staff have a duty of care to you and others around you. If they feel that there is a risk of significant harm to you or others, they will, wherever possible, discuss this with you and take action to ensure your safety.

**Who will be informed of my participation?**

Your GP will be notified that you are taking part in this study. Your GP may also receive information about your health from a therapist, for instance if they have concerns about your blood pressure. The therapist will discuss this with you first.

At the start of the study, we will ask you to nominate a ‘personal consultee’. This is someone who can make decisions about your participation in the study, should you no longer be able to do this yourself for any reason. The ‘personal consultee’ is often the same person who is taking part in the study with you, but you may prefer it to be someone else. They will only be consulted about your participation in the study if you are no longer able to make this decision for yourself.

**What will happen if I don’t want to carry on with the study?**

You are free to withdraw from your therapy at any time without giving any reason and without your legal rights being affected. We are interested in the reasons why people may choose to withdraw, so we will ask you about this if you are agreeable. If you withdraw only from the therapy, we would still like to collect research information. If you withdraw from the research study completely (both therapy and research), we will still use the information we have already collected in analyses, unless you ask us not to.

**What will happen to the results of this study?**

The information you provide will be anonymous and none of the participants will be identified in any report or publication. The results of the study may be published in medical journals and presented at scientific conferences. If you want to see the results, or the publication, please ask your researcher.

**Who is organising and funding this research?**

The research is being organised by Nottingham University Hospitals NHS Trust, and the University of Nottingham in collaboration with [Site Name]. The research is funded by the National Institute of Health Research (part of the NHS).

**Who has reviewed the study?**

All research in the NHS is looked at by an independent group of people called a Research Ethics Committee, to protect your interests. This study has been reviewed and given a favourable opinion by the Yorkshire & the Humber – Bradford-Leeds NHS Research Ethics Committee. The study has also been reviewed and approved by the Research & Innovation department of Nottingham University Hospitals NHS Trust.

**PRIVACY STATEMENT**

We will comply with the General Data Protection Regulation 2018. Information about your health is sensitive. We will hold and use the research information we collect from you and your medical records for medical and scientific research. We can use your information legally because a) it is used for scientific research b) it is used in the public interest c) you gave your permission.

We will protect your data using a number to identify you. Your name and address will be held separately and securely. We will use approved physical and computer security measures to keep it private. You have the right to ask us to show you the data we hold, and correct it if it is wrong. We will not use your data if you specifically ask us not to. We will not process the information automatically or use it for profiling. The information will be shared with the therapy team who see you, and the researchers who are collecting and analysing it. It may also be seen by people working for the Research Sponsor, or the Research Ethics Committee, who are responsible for ensuring that the study is carried out correctly. We will not transfer your information outside the European Union.

At the end of the study, your information will be kept securely for a minimum of 15 years. We will then arrange for confidential disposal. Research information can be useful to other researchers. The research funding body (NIHR) requires us to put your data in a store (the UK Data Archive), where others can use it, in an anonymised form, which means that no-one will be able to identify or contact you if they use it.

We will comply with University of Nottingham privacy policies, which can be found at https://www.nottingham.ac.uk/utilities/privacy/privacy.aspx

**Further information and contact details**

For further information in the first instance please contact:

[Site Contact Name

Job role

Address

Phone

Email]

For further information you may also contact the Chief Investigator of the study, Rowan Harwood:

Professor Rowan Harwood

School of Health Sciences University of Nottingham, and Nottingham University Hospitals NHS Trust, Queens Medical Centre, Nottingham NG7 2UH.

Phone: 0115 8230873

Email: rowan.harwood@nottingham.ac.uk

**Month**

**Therapy Package 1**

**Therapy Package 2**

Complete a daily calendar. Return via post once a month.

| Researcher visit (around 2.5 hours). Wear pedometer for 7 days. |  |  |  |
| --- | --- | --- | --- |
|  |  |  | Researcher visit (around 2.5 hours). Wear pedometer for 7 days. |
| Therapist visit (up to 3 visits) |  |  |  |
| Postal questionnaire to be completed by family member or friend. |  |  | Postal questionnaire to be completed by family member or friend. |
|  |  |  |  |
|  |  |  |  |
|  |  |  | Complete a daily calendar. Return via post once a month.  Therapists visit to deliver exercise and activity intervention (9 - 50 visits). |
|  |  |  |  |
|  |  |  |  |
|  |  |  |  |
|  |  |  |  |
|  |  |  | Researcher visit (around 2 hours). Wear pedometer for 7 days. |
| Researcher visit (around 2 hours).  Wear pedometer for 7 days. |  |  |  |
|  |  |  |  |
|  |  |  |  |
