# Supplementary material for: A randomised controlled trial of an exercise intervention promoting activity, independence and stability in older adults with mild cognitive impairment and early dementia (PrAISED) - A Protocol
Source: Trials. 2019 Dec 30;20:815. doi: 10.1186/s13063-019-3871-9 (PMC6937783; doi:10.1186/s13063-019-3871-9)
Supplement: Supplementary file 4 — Additional file 4. Summary participant information sheet. [file 13063_2019_3871_MOESM4_ESM.docx]

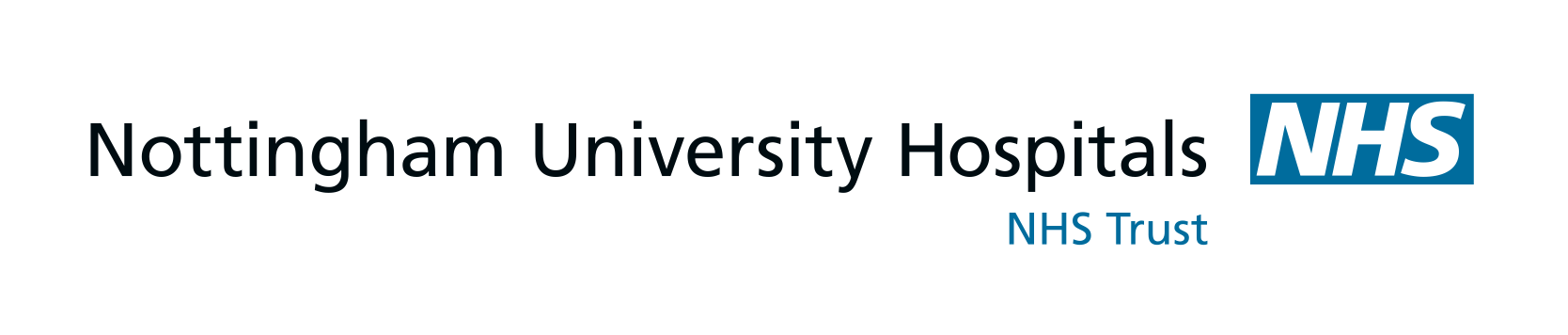

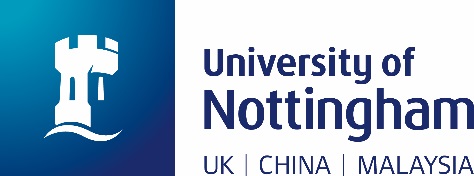


**Summary** **Participant Information Sheet - Patient**

**Promoting Activity, Independence and Stability in Early Dementia and Mild Cognitive Impairment (PrAISED 2)**

**Principal Investigator:** [Site PI name here]

**IRAS Project Identification Number:** 236099

**We would like to invite you to take part in a research study.**

People with memory problems can struggle with everyday activities, are more prone to accidents, and may stop doing things they want to do. We have designed a therapy package to help maintain activity, independence, and balance. We aim to test this through a ‘randomised controlled trial’.

**If you decide to take part:**

- We will visit you at home. We will ask you and a family member or carer questions about your health, wellbeing and the activities you do. This will take up to 2 hours.
- We will allocate you (at random) one of two therapy packages:
- Package 1: Assessment and advice with 1-3 visits from a therapist.
- Package 2: Assessment between 9 and 50 therapist visits over 12 months.
- We will ask you to record your exercises and falls in a daily calendar for 15 months.
- After 6 months, we will post out questionnaires for your carer to complete.
- We will visit you again after 12 months.
- We would like permission to examine your health and care records.
- We may want to video-record you during a session with a therapist.
- We may ask you to tell us what you thought about the therapy in an interview.

**You don’t have to take part, if you don’t want to.**

Please ask if you want more time to make up your mind, or if you need to know more. You can stop taking part at any time, just by telling us.

**Any information you give us will be kept confidential.**

Members of the research and therapy team will see your information, including your name and contact details. The information you provide for the research will be anonymous. This information will be recorded and stored separately from your name and contact details. No participants will be identified in any report or publication. We will tell your GP that you are taking part in the study. For more information on how your data will be processed please refer to the Participant information sheet – Patient.

**There should be very little risk from taking part in the study.**

The independent Yorkshire & The Humber; Bradford-Leeds NHS Research Ethics Committee has looked at the study and is happy to let us do it. If you need to know more, or have any questions or concerns about the study, please ask your researcher, [Site contact name/tel number]

| Researcher visit (around 2.5 hours). Wear pedometer for 7 days. |  |  |  |
| --- | --- | --- | --- |
|  |  |  | Researcher visit (around 2.5 hours). Wear pedometer for 7 days. |
| Therapist visit (up to 3 visits) |  |  |  |
| Postal questionnaire to be completed by family member or friend. |  |  | Postal questionnaire to be completed by family member or friend. |
|  |  |  |  |
|  |  |  |  |
| Complete a daily calendar. Return via post once a month. |  |  | Complete a daily calendar. Return via post once a month. |
|  |  |  |  |
|  |  |  |  |
|  |  |  | Therapists visit to deliver exercise and activity intervention (9 - 50 visits). |
|  |  |  |  |
|  |  |  |  |
| Researcher visit (around 2 hours). Wear pedometer for 7 days. |  |  | Researcher visit (around 2 hours). Wear pedometer for 7 days. |
|  |  |  |  |
|  |  |  |  |

**Month**

**Therapy Package 2**

**Therapy Package 1**
